# Evaluation of Knowledge and Attitude Among General Dental Practitioners Towards the Impact of Psychological Factors Affecting Periodontium: A Cross-Sectional Questionnaire Survey

**DOI:** 10.7759/cureus.110732

**Published:** 2026-06-12

**Authors:** Dhruvi R Patel, Rishabh Trivedi, Stuti Parikh, Pooja Gandhi, Brinda Vekariya

**Affiliations:** 1 General Dentistry, Private Practice, Vadodara, IND; 2 Periodontology, K. M. Shah Dental College and Hospital, Sumandeep Vidyapeeth (Deemed to be University), Vadodara, IND; 3 Dentistry, Government Dental College and Hospital, Jamnagar, IND; 4 Epidemiology and Public Health, Dentistry, The University of Texas Health Science Center at Houston, Houston, USA; 5 General Dentistry, Private Practice, Ottawa, CAN; 6 General Dentistry, Private Practice, Chicago, USA

**Keywords:** anxiety, behavioral factors, depression, general dental practitioners, oral health, periodontal health, periodontitis, psychological stress, psychosocial factors, stress management

## Abstract

Background

Periodontal disease is a multifactorial inflammatory condition influenced not only by microbial plaque but also by systemic, environmental, behavioral, and psychological factors. Psychological conditions, such as stress, anxiety, and depression, may contribute to periodontal disease progression through altered immune responses, increased inflammatory mediators, poor oral hygiene practices, smoking, and decreased compliance with dental treatment. General dental practitioners (GDPs) are often the first healthcare professionals to identify oral manifestations associated with psychological distress. Therefore, adequate knowledge and awareness regarding the relationship between psychological factors and periodontal health are essential for comprehensive patient management. However, limited data are available regarding the awareness and attitudes of GDPs toward the psychosocial aspects of periodontal disease.

Aim

The present study aimed to evaluate the knowledge, attitudes, and clinical practices of GDPs regarding the impact of psychological factors on periodontal health.

Materials and methods

A descriptive cross-sectional questionnaire-based survey was conducted among GDPs. A structured self-administered questionnaire consisting of demographic details, knowledge-based questions, attitude assessment, and clinical practice-related questions was developed after reviewing relevant literature. The questionnaire was validated by subject experts and pilot-tested prior to circulation. The final questionnaire was distributed electronically using Google Forms. Responses obtained were compiled and analyzed using descriptive statistical methods. Frequencies and percentages were calculated for categorical variables.

Results

Most participants demonstrated awareness regarding the influence of psychological factors on periodontal health. Stress and anxiety were the most commonly identified by practitioners as being linked to periodontal disease progression. A majority of respondents agreed that psychological evaluation should be incorporated into routine dental examinations; however, fewer practitioners reported routinely discussing psychological well-being with patients. Participants showed moderate understanding regarding mechanisms linking psychological stress and periodontal disease, including altered immune response, behavioral changes, and reduced treatment compliance. Limited confidence in managing stress-related periodontal conditions and a lack of formal training were identified as common barriers among practitioners.

Conclusion

GDPs demonstrated a positive attitude and moderate level of awareness regarding the influence of psychological factors on periodontal health. However, gaps remain in practical implementation and confidence in addressing psychosocial concerns during routine periodontal care. Incorporating behavioral sciences and psychosocial assessment training into undergraduate dental education and continuing professional development programs may improve holistic patient management and periodontal treatment outcomes.

## Introduction

Periodontal disease is a chronic multifactorial inflammatory condition affecting the supporting structures of teeth, including the gingiva, periodontal ligament, cementum, and alveolar bone [1]. Although microbial dental plaque is considered the primary etiological factor, the progression and severity of periodontal disease are significantly influenced by systemic, behavioral, environmental, and psychosocial factors [2]. In recent years, increasing evidence has highlighted the role of psychological factors such as stress, anxiety, depression, and emotional disturbances in influencing periodontal health and treatment outcomes [3].

Psychological stress may alter immune and inflammatory responses through activation of the hypothalamic-pituitary-adrenal axis and sympathetic nervous system. Increased cortisol secretion associated with chronic stress can suppress immune function, impair wound healing, and increase susceptibility to periodontal destruction [4,5]. Furthermore, stress-related elevations in inflammatory mediators, such as interleukin-1β and tumor necrosis factor-alpha, have been associated with periodontal tissue breakdown [6].

Apart from biological mechanisms, psychological factors can indirectly affect periodontal health through behavioral changes. Individuals experiencing stress or depression often demonstrate poor oral hygiene maintenance, irregular dental visits, tobacco consumption, unhealthy dietary practices, bruxism, and reduced compliance with periodontal therapy [7]. These factors may accelerate plaque accumulation and worsen periodontal outcomes. Depression has additionally been associated with increased periodontal attachment loss and gingival inflammation due to decreased motivation toward self-care and preventive dental practices [8].

The concept of psychoneuroimmunology has enhanced understanding regarding the interaction between psychological processes, neurological responses, endocrine pathways, and immune modulation in chronic inflammatory diseases [9]. Modern periodontal therapy therefore emphasizes a holistic approach that considers psychosocial well-being alongside conventional clinical parameters.

General dental practitioners (GDPs) are often the first healthcare professionals to identify oral manifestations associated with psychological distress, including stress-related periodontal inflammation, bruxism, xerostomia, and poor oral hygiene maintenance [10]. Awareness regarding psychosocial determinants of periodontal disease is therefore important for early diagnosis, patient counseling, interdisciplinary referral, and comprehensive periodontal care.

Despite growing scientific evidence supporting the relationship between psychological stress and periodontal disease, awareness among dental practitioners remains variable. Limited literature is available regarding the knowledge, attitudes, and clinical practices of GDPs concerning psychological influences on periodontal health, especially in the Indian population [11]. Therefore, the present cross-sectional questionnaire-based survey was conducted to evaluate the knowledge, attitudes, and clinical practices of GDPs regarding the impact of psychological factors on periodontal health.

## Materials and methods

Study design

This cross-sectional, questionnaire-based descriptive study was conducted to evaluate the knowledge, attitudes, and clinical practices of dental practitioners regarding the impact of psychological factors on periodontal health. The study was conducted in accordance with the principles of the Declaration of Helsinki. Before answering the survey, all participants were presented with an electronic informed consent form outlining the study's purpose, ensuring anonymity, and stating that participation was entirely voluntary.

Study population and eligibility criteria

The target population consisted of practicing dental professionals. To be included in the study, respondents had to be registered dental practitioners (including general dentists and dental specialists) who actively manage patients with periodontal conditions. Dental students, non-practicing professionals, and individuals who did not provide informed consent were excluded from the analysis.

A non-probability convenience sampling strategy was utilized to recruit participants. The minimum sample size required was determined using the standard formula for cross-sectional studies:

$n = \frac{Z^2 \cdot p \cdot (1 - p)}{d^2}$

Here, Z is the standard normal deviation at a 95% confidence interval (1.96), p is the expected proportion of awareness (assumed to be 50% or 0.5 to maximize sample size), and d is the margin of error (5% or 0.05). This yielded a minimum sample size requirement of approximately 384 participants. A final sample of 404 completed responses was gathered and included in the final data analysis.

Data collection instrument (questionnaire)

Data were collected using a structured, closed-ended electronic questionnaire developed via Google Forms (Alphabet Inc., Mountain View, CA). The questionnaire was distributed electronically through professional dental networks, social media platforms (e.g., WhatsApp, LinkedIn), and email registries to ensure broad reach. The survey instrument was adapted from previous literature and structured into four distinct domains:

Demographic and Professional Profile

Questions regarding years of clinical experience (< 1 year, 1-5 years, 6-10 years, and > 10 years) and primary area of practice (general dentistry, periodontics, prosthodontics, oral and maxillofacial surgery, endodontics, orthodontics, and pedodontics).

Knowledge and Self-Perception

Items evaluating general awareness of the link between psychological factors and periodontal health, along with a self-rated confidence score measured on a categorical scale (very confident, somewhat confident, neutral, not confident at all).

Perceived Mechanisms and Risk Factors

Questions exploring specific psychological modifiers (stress, anxiety, depression, insomnia, emotional exhaustion) and perceived pathophysiological or behavioral pathways (e.g., neglect of oral hygiene, suppressed immune response, increased inflammation, altered salivary composition).

Clinical Attitudes and Practice Behaviors

Items detailing patient counseling tendencies, preferred patient education modalities, behavioral goals, clinical treatment modifications, and interdisciplinary referral frequencies to mental health professionals.

To ensure face and content validity, the questionnaire was reviewed by a panel of dental public health and periodontal experts. A pilot study was conducted on a small cohort of dental practitioners (n = 20, excluded from the final analysis) to verify clarity, comprehension, and the internal consistency of the questions before formal distribution.

Statistical analysis

Data were extracted from Google Forms (Alphabet Inc., Mountain View, CA), cleaned, and tabulated. Statistical analysis was performed using Statistical Product and Service Solutions (SPSS, version 21.0; IBM SPSS Statistics for Windows, Armonk, NY).

Descriptive statistics were applied to summarize data, where categorical variables (e.g., demographics, knowledge levels, and clinical behaviors) were expressed as absolute frequencies (n) and relative percentages (%). For multi-select questions, percentages were computed based on the total number of unique respondents (N = 404).

To evaluate whether the observed distribution of responses within single-choice categorical variables significantly deviated from an expected equal distribution (where choices are selected with equal probability), a chi-square goodness-of-fit test was performed. The degrees of freedom (df) were calculated based on the number of response categories minus one (k - 1). For all statistical tests, a two-tailed p-value of less than 0.05 (p < 0.05) was considered statistically significant.

## Results

Demographic and professional characteristics of respondents

**Figure 1:**
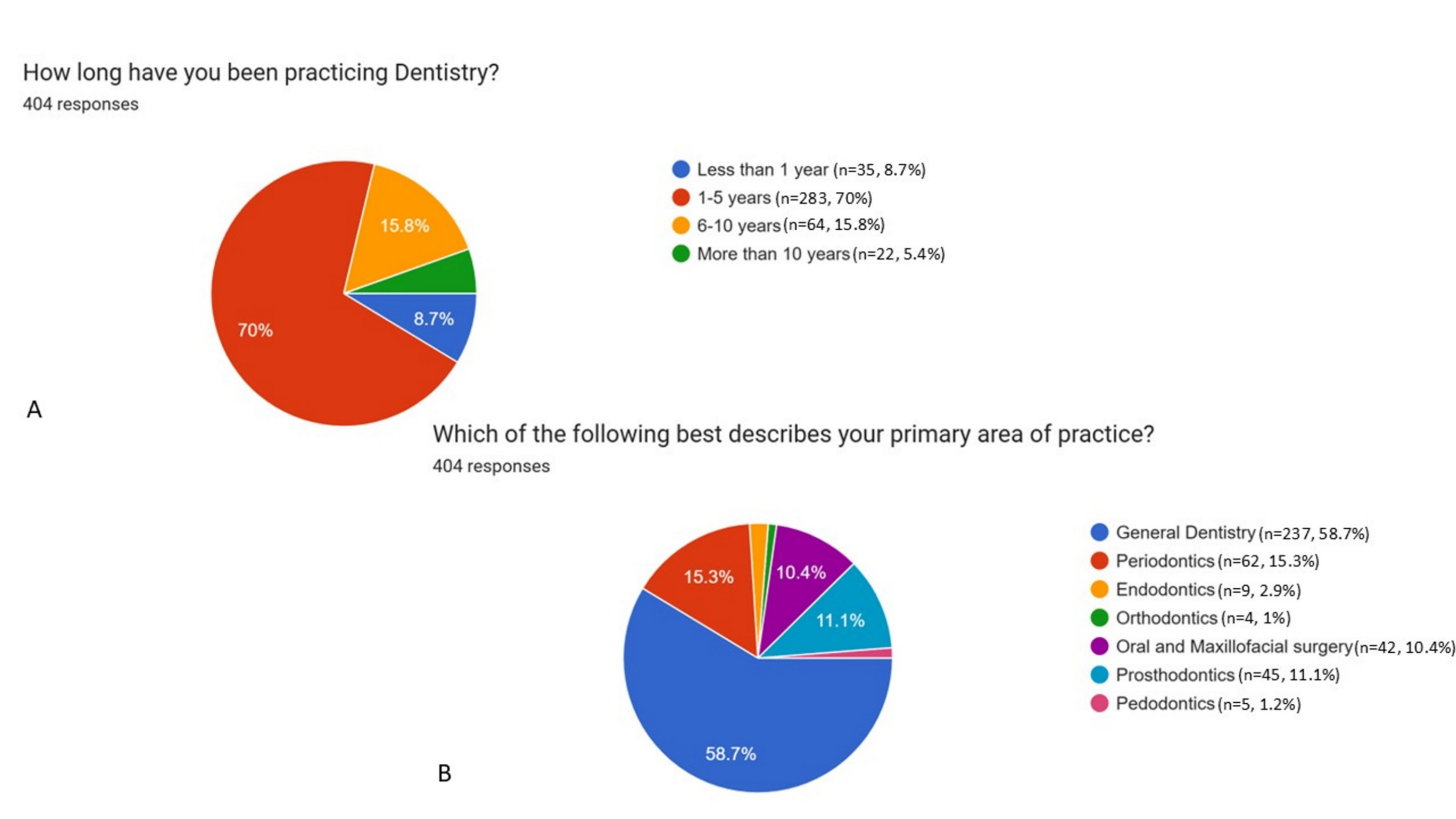
Basic Information A: How long have you been practicing dentistry? B: Which of the following best describes your primary area of practice?

**Figure 2:**
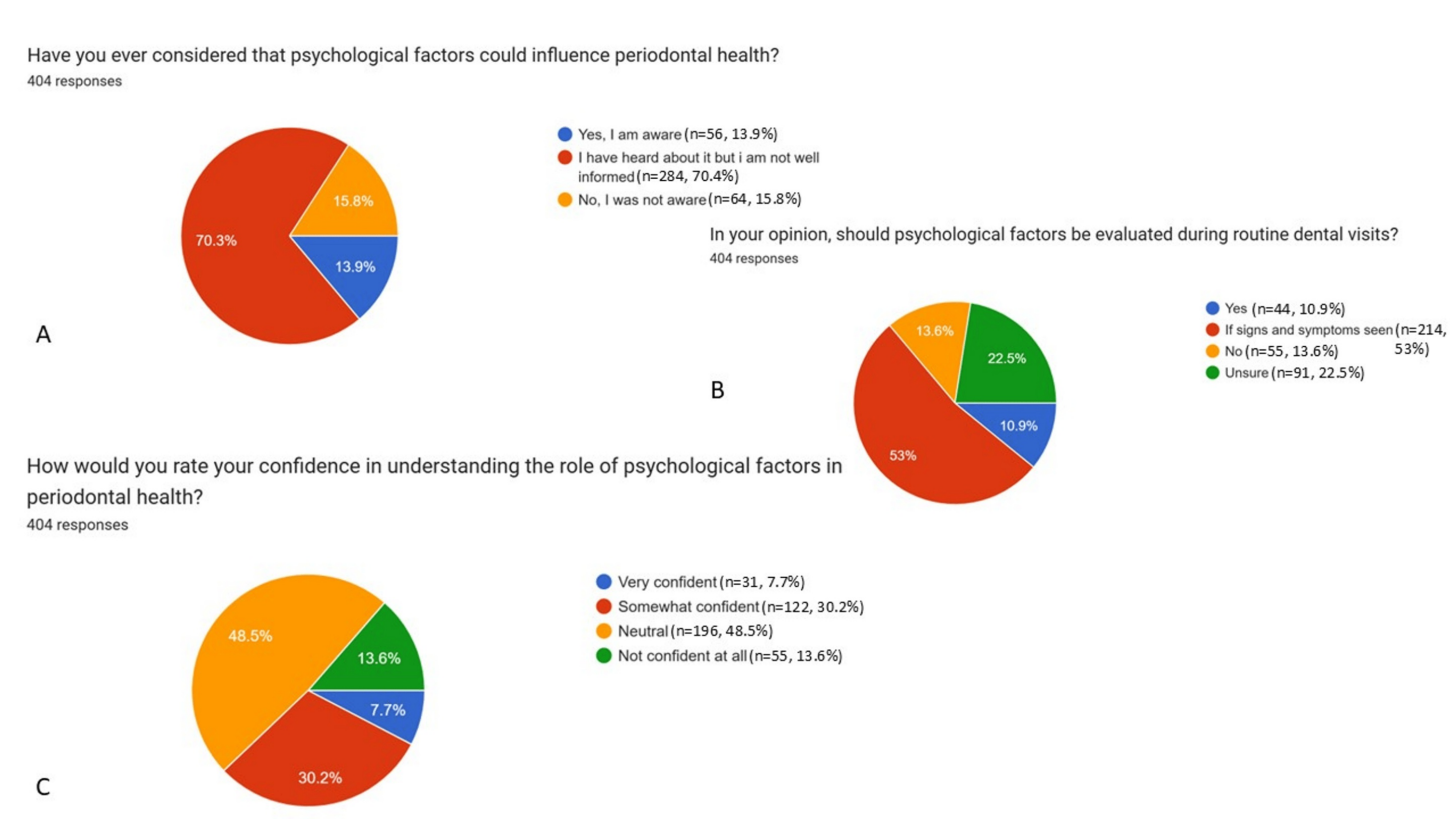
Knowledge, Awareness, and Perception About Clinical Aspects (Part 1) A: Have you ever considered that psychological factors could influence periodontal health? B: In your opinion, should psychological factors be evaluated during routine dental visits? C: How would you rate your confidence in understanding the role of psychological factors in periodontal health?

A total of 404 dental practitioners completed the survey. The respondents' professional profiles included years of clinical experience and primary areas of dental practice, as cross-referenced in Table 1 and visually outlined in Figures 1-2.

**Table 1:** Demographic and Professional Characteristics of Respondents (N = 404) Statistically significant at p < 0.001

Variable	Category	Frequency (n)	Percentage (%)	Statistical Analysis
Years of Practice	Less than 1 year	35	8.70%	X^2^=485.43, df=3, p<0.001
	1-5 years	283	70.00%	
	6-10 years	64	15.80%	
	More than 10 years	22	5.50%	
Primary Area of Practice	General Dentistry	237	58.70%	X^2^=603.54, df=6, p<0.001
	Periodontics	62	15.30%	
	Prosthodontics	45	11.10%	
	Oral & Maxillofacial Surgery	42	10.40%	
	Endodontics	6	1.50%	
	Orthodontics	6	1.50%	
	Pedodontics	6	1.50%	

The majority of respondents had been practicing dentistry for one to five years (70.0%, n = 283), followed by 6-10 years (15.8%, n = 64), less than one year (8.7%, n = 35), and more than 10 years (5.5%, n = 22) (Figure 1). A chi-square goodness-of-fit test demonstrated that this distribution was significantly unequal (X^2^(3) = 485.43, p < 0.001; Table 1).

Regarding the primary area of practice, general dentistry constituted the largest segment (58.7%, n = 237), followed by periodontics (15.3%, n = 62), prosthodontics (11.1%, n = 45), and oral and maxillofacial surgery (10.4%, n = 42) (Figure 2). The remaining practitioners were distributed among endodontics, orthodontics, and pedodontics (combined 4.5%, n = 18). This distribution was also highly statistically significant (X^2^(6) = 603.54, p < 0.001; Table 1).

Knowledge and perceptions of psychological factors in periodontal health

The survey evaluated practitioners' awareness, clinical opinions, and self-rated confidence regarding the interplay between mental health and periodontal diseases (Table 2).

**Table 2:** Knowledge, Opinions, and Self-Rated Confidence (N = 404) Statistically significant at p < 0.001.

Survey Item	Response Category	Frequency (n)	Percentage (%)	Statistical Analysis
Considered psych factors	Yes, I am aware	56	13.90%	X^2^=314.54, df=2, p<0.001
	Heard about it, not well informed	284	70.30%	
	No, I was not aware	64	15.80%	
Evaluate during routine visits	Yes	44	10.90%	X^2^=179.86, df=3, p<0.001
	If signs and symptoms seen	214	53.00%	
	No	55	13.60%	
	Unsure	91	22.50%	
Confidence rating	Very confident	31	7.70%	X^2^=145.47, df=3, p<0.001
	Somewhat confident	122	30.20%	
	Neutral	196	48.50%	
	Not confident at all	55	13.60%	

When asked if they considered that psychological factors could influence periodontal health, a substantial majority reported being aware of the connection but not well informed (70.3%, n = 284). Only 13.9% (n = 56) claimed to be fully aware, while 15.8% (n = 64) were not aware (X^2^(2) = 314.54, p < 0.001; Table 2).

Regarding clinical practices, 53.0% (n = 214) believed psychological factors should be evaluated during routine dental visits only if signs and symptoms are seen. Meanwhile, 22.5% (n = 91) were unsure, 13.6% (n = 55) answered "No", and only 10.9% (n = 44) supported routine evaluations for all patients (X^2^(3) = 179.86, p < 0.001; Table 2).

Practitioners' confidence in understanding this relationship matched their self-declared knowledge deficits: 48.5% (n = 196) rated their confidence as neutral, 30.2% (n = 122) felt somewhat confident, 13.6% (n = 55) were not confident at all, and only 7.7% (n = 31) felt very confident (X^2^(3) = 145.47, p < 0.001; Table 2).

Perceived impacting factors and biological mechanisms

**Figure 3:**
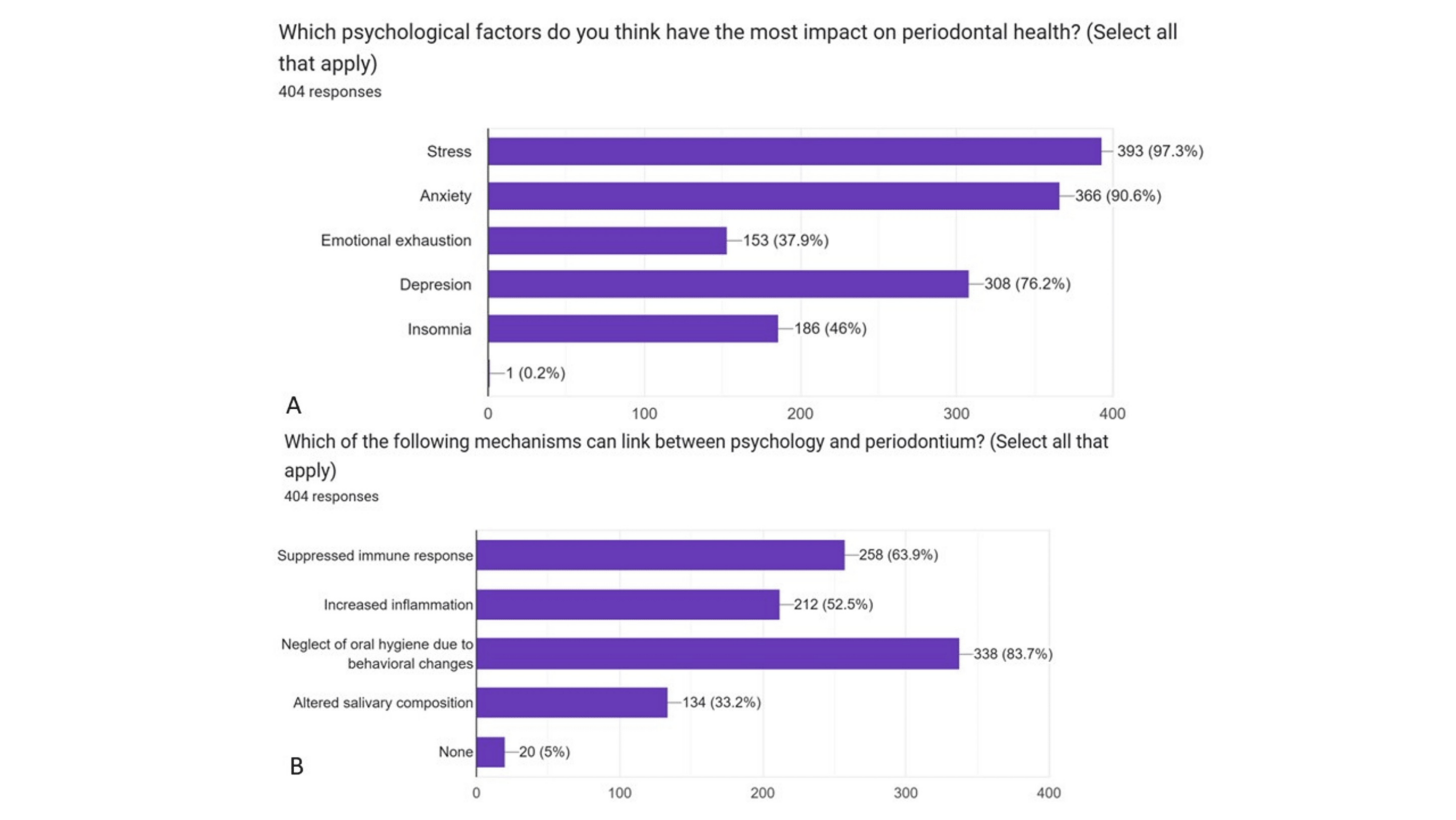
Knowledge, Awareness, and Perception about Clinical Aspects (Part 2) A: Which psychological factors do you think have the most impact on periodontal health? (Select all that apply). B: Which of the following mechanisms can link between psychology and periodontium? (Select all that apply)

**Figure 4:**
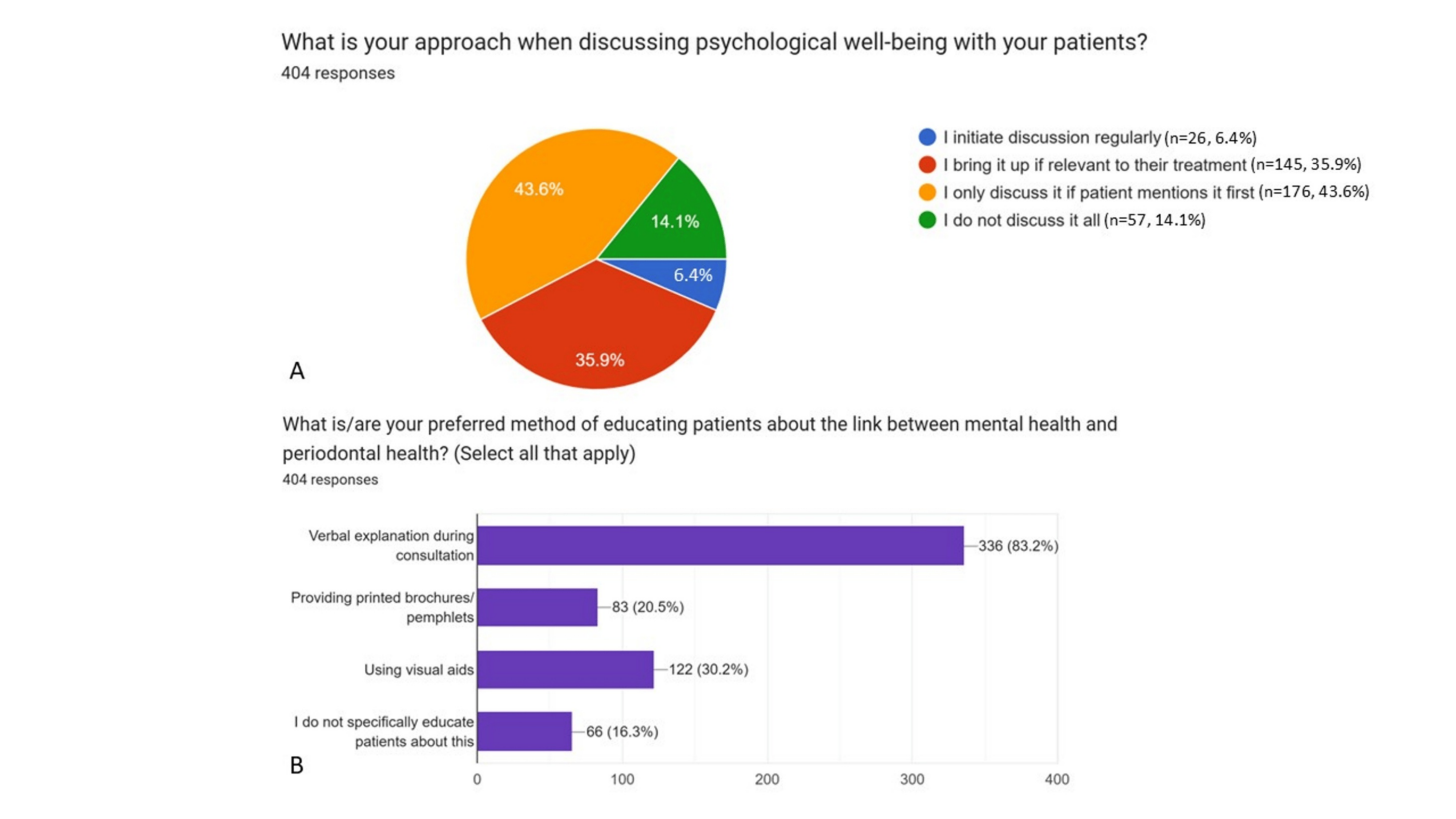
Knowledge, Awareness, and Perception About Clinical Aspects (Part 3) A: What is your approach when discussing psychological well-being with your patients? B: What is/are your preferred method of educating patients about the link between mental health and periodontal health? (Select all that apply)

Respondents were asked to select multiple options regarding which specific psychological conditions affect periodontal tissue and through which biological pathways (Table 3, Figures 3-4).

**Table 3:** Perceived Impacting Factors and Pathogenic Mechanisms (Multiple Choice)

Variable (Select all that apply)	Category Selection	Frequency (n)	Selection % (N=404)
Psychological factors	Stress	393	97.30%
	Anxiety	366	90.60%
	Depression	308	76.20%
	Insomnia	186	46.00%
	Emotional exhaustion	153	37.90%
	Other	1	0.20%
Linking mechanisms	Neglect of oral hygiene (behavioral)	338	83.70%
	Suppressed immune response	258	63.90%
	Increased inflammation	212	52.50%
	Altered salivary composition	134	33.20%
	None	20	5.00%

Stress was almost universally recognized as an impacting factor (97.3%, n = 393), closely followed by anxiety (90.6%, n = 366) and depression (76.2%, n = 308). Insomnia (46.0%, n = 186) and emotional exhaustion (37.9%, n = 153) were noted less frequently (Figure 3).

In terms of the physiological and behavioral mechanisms linking psychology to the periodontium, neglect of oral hygiene due to behavioral changes was selected most frequently (83.7%, n = 338). Biological pathways were also recognized, including a suppressed immune response (63.9%, n = 258) and increased inflammation (52.5%, n = 212), while altered salivary composition was selected by 33.2% (n = 134) (Table 3).

Patient communication, clinical interventions, and referrals

**Figure 5:**
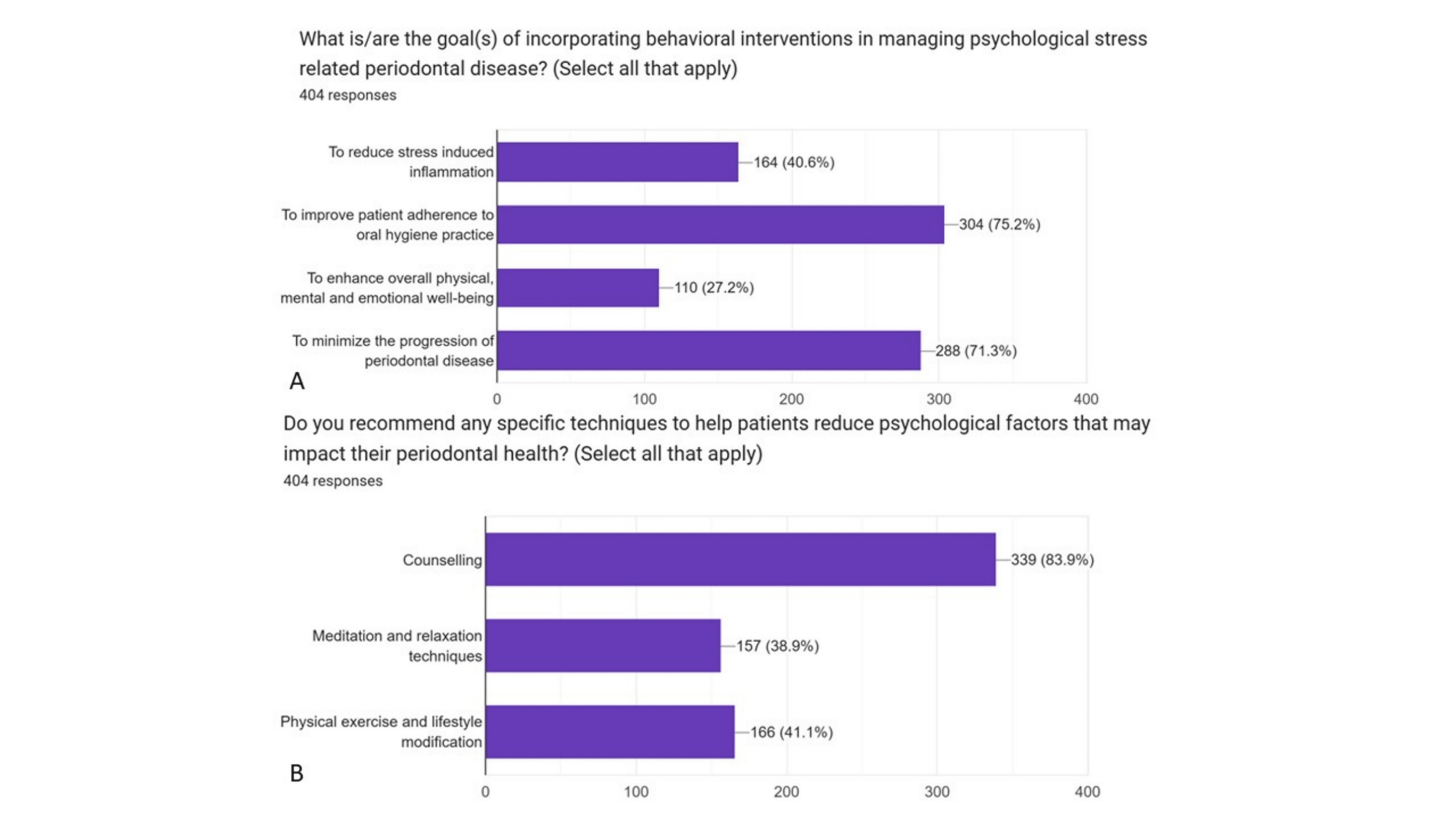
Awareness and Attitude Towards Management (Part 1) A: What is/are the goal(s) of incorporating behavioral interventions in managing psychological stress related periodontal disease? (Select all that apply). B: Do you recommend any specific techniques to help patients reduce psychological factors that may impact their periodontal health? (Select all that apply)

**Figure 6:**
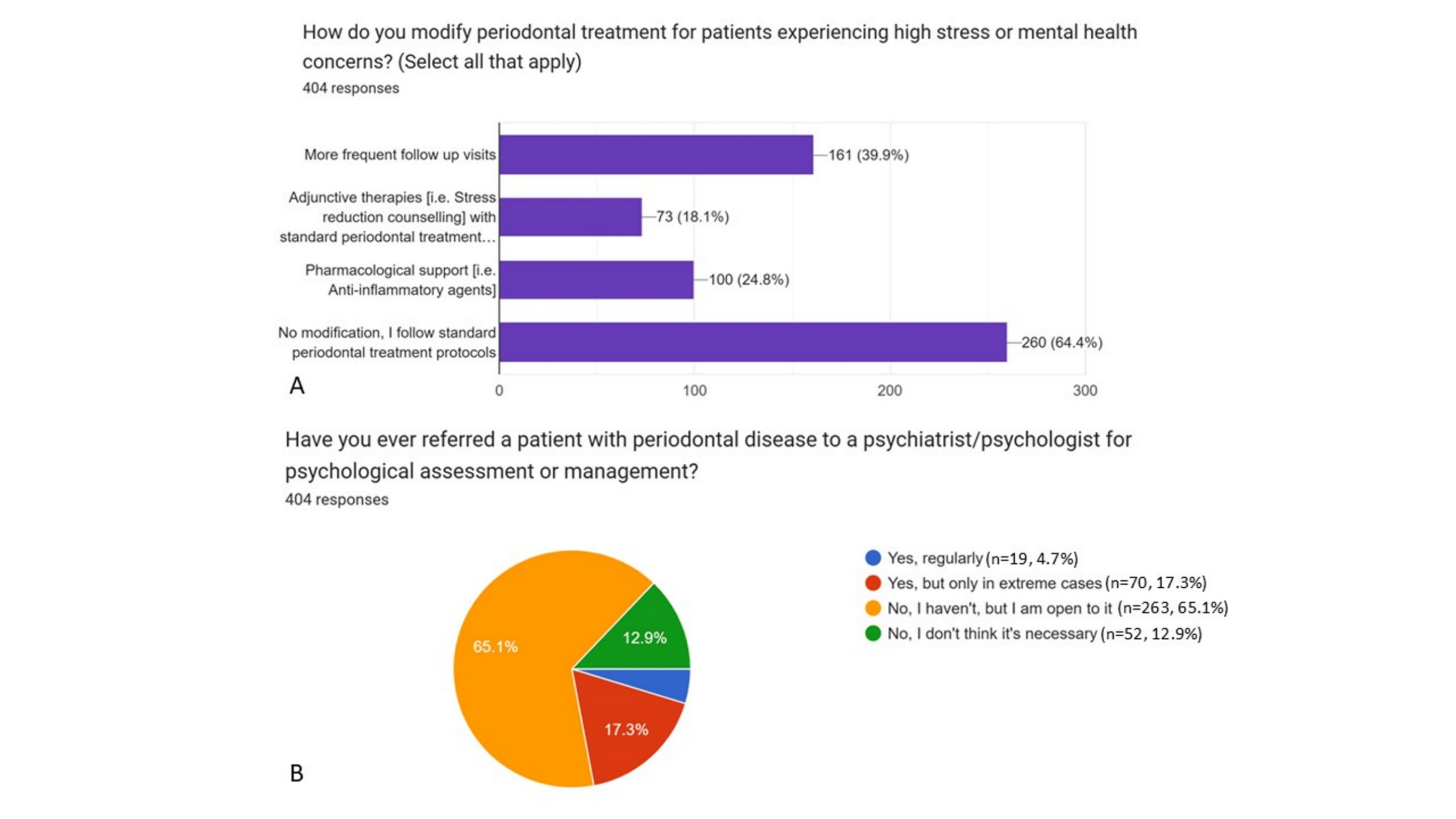
Awareness and Attitude Towards Management (Part 2) A: How do you modify periodontal treatment for patients experiencing high stress or mental health concerns? (Select all that apply). B: Have you ever referred a patient with periodontal disease to a psychiatrist/psychologist for psychological assessment or management?

Clinical behavior data indicated a predominantly reactive approach to addressing mental wellness with dental patients (Table 4, Figures 5-6).

**Table 4:** Clinical Approaches, Patient Education, and Referral Tendencies Statistically significant at p < 0.001.

Variable	Response Category/Choice	Frequency (n)	Percentage (%)	Statistical Analysis
Approach to discussing well-being	I initiate discussion regularly	19	4.70%	χ2=152.02, df=3, p<0.001
	I bring it up if relevant to treatment	145	35.90%	
	I only discuss if patient mentions it	176	43.60%	
	I do not discuss it at all	57	14.10%	
Preferred education method (multiple choice)	Verbal explanation during consultation	336	83.20%	N/A (Multi-select)
	Using visual aids	122	30.20%	
	Providing printed brochures/pamphlets	83	20.50%	
	I do not specifically educate patients	66	16.30%	
Intervention goal (multiple choice)	Improve patient adherence to hygiene	304	75.20%	N/A (Multi-select)
	Minimize progression of perio disease	288	71.30%	
	Reduce stress-induced inflammation	164	40.60%	
	Enhance overall physical/mental well-being	110	27.20%	
Recommended techniques (multiple choice)	Counselling	339	83.90%	N/A (Multi-select)
	Physical exercise/lifestyle modification	166	41.10%	
	Meditation and relaxation techniques	157	38.90%	
Treatment modification (multiple choice)	No modification, standard protocols	260	64.40%	N/A (Multi-select)
	More frequent follow-up visits	161	39.90%	
	Pharmacological support	100	24.80%	
	Adjunctive therapies (e.g., counselling)	73	18.10%	
Psychiatric/psychological referral	Yes, regularly	19	4.70%	χ2=288.63, df=3, p<0.001
	Yes, but only in extreme cases	70	17.30%	
	No, haven't but open to it	263	65.10%	
	No, don't think it is necessary	52	12.90%	

Only 4.7% (n = 19) of dentists stated they regularly initiate discussions regarding psychological well-being. The majority preferred to discuss it only if the patient mentions it first (43.6%, n = 176) or if it is directly relevant to treatment (35.9%, n = 145). A small cohort (14.1%, n = 57) avoids discussing it entirely (X^2^(3) = 152.02, p < 0.001; Table 4).

Verbal explanation during consultation was the primary method used to educate patients (83.2%, n = 336), with visual aids (30.2%, n = 122) and printed materials (20.5%, n = 83) used significantly less (Table 4). Notably, 16.3% (n = 66) do not explicitly educate patients on this topic.

When managing stress-related periodontal diseases, improving patient adherence to oral hygiene practices was the most frequently endorsed goal of behavioral interventions (75.2%, n = 304), followed by minimizing disease progression (71.3%, n = 288). Counseling was the most widely recommended patient stress-reduction technique (83.9%, n = 339).

In terms of clinical treatment modifications, 64.4% (n = 260) follow standard protocols with no modifications for high-stress patients, while 39.9% (n = 161) schedule more frequent follow-up visits. Finally, interdisciplinary cross-referral remains uncommon (Figure 6): 65.1% (n = 263) of practitioners have never referred a periodontal patient to a mental health professional (though they remain open to it), and 12.9% (n = 52) deem it completely unnecessary (X^2^(3) = 288.63, p < 0.001; Table 4).

## Discussion

The present study offers a comprehensive evaluation of dental practitioners' knowledge, attitudes, and clinical behaviors regarding the bidirectional relationship between psychological factors and periodontal disease. Periodontitis is recognized as a complex, multifactorial inflammatory disease driven by a dysbiotic microbial biofilm and modulated by host immune responses [1]. While traditional risk factors, such as smoking and diabetes, are routinely integrated into clinical assessments [2], the influence of psychological modifiers - such as stress, anxiety, and depression - remains frequently overlooked in active day-to-day dental practice, despite robust evidence validating their impact [3,4].

Interpretation of knowledge and awareness deficits

Our results demonstrate a critical paradox in the dental community: while 84.2% of surveyed practitioners acknowledged an awareness of the psychological-periodontal link (either fully aware or having heard about it), a striking 70.3% admitted they were "not well informed." This pervasive self-declared knowledge deficit aligns closely with the fact that nearly half of the cohort (48.5%) expressed a strictly neutral stance regarding their confidence in managing these factors.

This finding is highly relevant when viewed alongside established literature. Classic and contemporary studies have thoroughly documented that chronic psychological stressors and depressive states significantly accelerate periodontal tissue destruction [5,6]. The widespread lack of structural, detailed knowledge among our respondents suggests that undergraduate and continuing dental education curricula may not sufficiently emphasize the clinical implications of psychoneuroimmunology in oral health.

Mechanisms: behavioral changes vs. pathophysiological pathways

When identifying the primary mechanisms bridging mental wellness and periodontal disease, our respondents prioritized behavioral alterations over biological ones. A majority (83.7%) selected the neglect of oral hygiene due to psychological distress as a major pathway. This matches established behavioral models showing that high-stress or depressed states frequently lead to a cessation of self-care routines, increased substance abuse, and poor dietary choices [7,8].

However, a lower proportion of practitioners selected biological mechanisms such as a suppressed immune response (63.9%), increased systemic/local inflammation (52.5%), or altered salivary composition (33.2%). This relative omission is concerning because the biological pathways are profoundly destructive. Chronic stress triggers the activation of the hypothalamic-pituitary-adrenal (HPA) axis and the sympathetic nervous system [9]. This neuroendocrine activation elevates systemic cortisol and catecholamines, which directly compromise host immune defenses, facilitate local microbial dysbiosis, and upregulate pro-inflammatory cytokines like interleukin-6 (IL-6) in the gingival crevicular fluid [10-12].

The fact that our respondents underestimated these systemic mechanisms suggests that many dentists still view psychological distress primarily as a behavioral hurdle rather than an organic, pathophysiological modifier of tissue biology.

Reactive clinical practices and barriers to care

The clinical behavior data collected in this study reflect a heavily reactive, rather than proactive, approach to patient care. More than half of the practitioners (53.0%) believed that psychological parameters should only be evaluated if obvious signs or symptoms are visually apparent, and a combined 79.5% only discuss psychological well-being if the patient brings it up first or if it becomes strictly necessary for treatment logistics.

This hesitation to initiate discussions regarding mental wellness creates an invisible barrier to comprehensive care. Literature emphasizes that patients frequently compartmentalize dental appointments away from their psychological struggles due to fear, perceived stigma, or a belief that their dentist lacks interest in their mental state [13,14]. When dentists avoid initiating these conversations, opportunities for early risk modification are entirely lost.

Furthermore, while 83.2% of our respondents utilize verbal explanations during consultations to educate patients, 16.3% do not explicitly educate patients on the link between stress and oral health at all. This highlights a missed opportunity for comprehensive patient education, especially considering how anxiety and dental pain perceptions can negatively feed into each other, further worsening patient compliance and treatment success [15,16].

Interdisciplinary referrals and treatment modifications

Perhaps the most definitive finding regarding current clinical inertia is that 65.1% of surveyed practitioners have never referred a periodontal patient to a mental health professional, despite claiming an openness to do so, while 12.9% deemed such referrals entirely unnecessary. Interdisciplinary cross-referral remains exceptionally rare in dentistry [17]. This occurs despite explicit evidence that managing systemic stress and utilizing targeted psychological interventions can successfully yield improvements in clinical attachment levels and reduced gingival inflammation [18].

While most dentists in our sample rely on standard clinical protocols without modifications (64.4%), a subset did note utilizing behavioral support mechanisms, such as emphasizing oral hygiene compliance (75.2%) or suggesting lifestyle modifications and relaxation techniques (41.1%). However, without a formalized, multidisciplinary referral pathway connecting dental clinics to psychological counseling services, these recommendations may carry limited weight.

Study limitations

This study has certain limitations. The cross-sectional design prevents the inference of causality regarding practitioner habits over time. The use of convenience sampling may introduce selection bias, as younger professionals (one to five years of experience represented 70.0% of our sample, or general dentists might be over-represented compared to long-standing specialists. Future multi-center studies utilizing random sampling are needed to confirm these trends globally.

## Conclusions

While a vast majority of dental practitioners recognize a conceptual link between psychological health and periodontal disease, an overwhelming majority remain under-informed and lack the confidence to manage these factors clinically. Practitioners tend to prioritize the behavioral consequences of psychological distress (e.g., oral hygiene neglect) over its direct, destructive pathophysiological effects on the host immune response and inflammatory pathways. Consequently, clinical interventions remain highly reactive, patient education is inconsistent, and interdisciplinary referrals to mental health professionals are rarely executed.

To bridge this gap, modern dental education and professional frameworks must shift from an isolated focus on local biofilms to a holistic, biopsychosocial approach. Integrating standardized psychological screening tools into routine periodontal evaluations, providing structured continuing education on psychoneuroimmunology, and establishing clearer cross-referral pathways between dental and mental healthcare fields are essential steps to improve long-term periodontal treatment outcomes and overall patient well-being.

## Appendices

Questionnaire survey

* Indicates required question

Basic information

1. How long have you been practicing dentistry?* (Mark only one)

Less than 1 year

1-5 years

6-10 years

More than 10 years

2. Which of the following best describes your primary area of practice?* (Mark only one)

General Dentistry

Periodontics

Endodontics

Orthodontics

Oral and maxillofacial surgery

Prosthodontics

Pedodontics

Knowledge, awareness, and perception about clinical aspects

3. Have you ever considered that psychological factors could influence periodontal health?* (Mark only one)

Yes, I am aware

I have heard about it, but I am not well-informed

No, I was not aware

4. In your opinion, should psychological factors be evaluated during routine dental visits?* (Mark only one)

Yes

If signs and symptoms seen

No

Unsure

5. How would you rate your confidence in understanding the role of psychological factors in periodontal health?* (Mark only one)

Very confident

Somewhat confident

Neutral

Not confident at all

6. Which psychological factors do you think have the most impact on periodontal health?* (Select all that apply)

Stress

Anxiety

Emotional exhaustion

Depresion

Insomnia

Other:

7. Which of the following mechanisms can link between psychology and periodontium?* (Select all that apply)

Suppressed immune response

Increased inflammation

Neglect of oral hygiene due to behavioral changes

Altered salivary composition

None

8. What is your approach when discussing psychological well-being with your patients?* (Mark only one)

I initiate discussion regularly

I bring it up if relevant to their treatment

I only discuss it if the patient mentions it first

I do not discuss it all

9. What is/are your preferred method of educating patients about the link between mental health and periodontal health?* (Select all that apply)

Verbal explanation during consultation

Providing printed brochures/pamphlets

Using visual aids

I do not specifically educate patients about this

Awareness and attitude towards management

10. What is/are the goal(s) of incorporating behavioral interventions in managing psychological stress-related periodontal disease?* (Select all that apply)

To reduce stress induced inflammation

To improve patient adherence to oral hygiene practice

To enhance overall physical, mental and emotional well-being

To minimize the progression of periodontal disease

11. Do you recommend any specific techniques to help patients reduce psychological factors that may impact their periodontal health?* (Select all that apply)

Counselling

Meditation and relaxation techniques

Physical exercise and lifestyle modification

Other:

12. How do you modify periodontal treatment for patients experiencing high stressor mental health concerns?* (Select all that apply)

More frequent follow-up visits

Adjunctive therapies (i.e. Stress reduction counselling) with standard periodontal treatment protocols

Pharmacological support (i.e. Anti-inflammatory agents)

No modification, I follow standard periodontal treatment protocols

13. Have you ever referred a patient with periodontal disease to a psychiatrist/psychologist for psychological assessment or management?* (Mark only one)

Yes, regularly

Yes, but only in extreme cases

No, I haven't, but I am open to it

No, I don't think it's necessary
